# Case report: Echocardiographic diagnosis of cardiac involvement caused by congenital generalized lipodystrophy in an infant

**DOI:** 10.3389/fped.2023.1087833

**Published:** 2023-03-23

**Authors:** Jie Zhou, Hanmin Liu, Jiao Chen, Xiaolan He

**Affiliations:** ^1^ Department of Ultrasonic Medicine, West China Second University Hospital of Sichuan University, Chengdu, China; ^2^ Key Laboratory of Birth Defects and Related Diseases of Women and Children (Sichuan University), Ministry of Education, Chengdu, China; ^3^ Department of Pediatric Pulmonology and Immunology, West China Second University Hospital of Sichuan University, Chengdu, China; ^4^ Key Laboratory of Chronobiology (Sichuan University), National Health Commission of China, Chengdu, China; ^5^ The Joint Laboratory for Lung Development and Related Diseases of West China Second University Hospital, Sichuan University and School of Life Sciences of Fudan University, West China Institute of Women and Children's Health, West China Second University Hospital of Sichuan University, Chengdu, China; ^6^ Sichuan Birth Defects Clinical Research Center, West China Second University Hospital of Sichuan University, Chengdu, China; ^7^ Tibet Autonomous Region Women's and Children's Hospital, West China Second University Hospital of Sichuan University, Lhasa, China; ^8^ Ziyang Maternal and Child Health Care Hospital, Ziyang, China; ^9^ Ziyang Women and Children Hospital, West China Second University Hospital of Sichuan University, Ziyang, China

**Keywords:** congenital generalized lipodystrophy, cardiac involvement, echocardiography, infant, systolic dysfunction

## Abstract

We herein first report the use of conventional echocardiography combined with two-dimensional speckle-tracking to diagnose and monitor the changing process of cardiac involvement in an infant with congenital lipodystrophy. An 8-month-old girl was admitted to our hospital after first presenting at the age of 3 months with abnormal facial features that had been noticed within 4 weeks of birth. Echocardiography performed at the age of 3 months showed only slightly accelerated blood flow in the right ventricular outflow tract. At the age of 5 months, echocardiography showed myocardial hypertrophy; this finding combined with the physical characteristics and other examination results led to the consideration of congenital lipodystrophy. Genetic testing at the age of 9 months confirmed type 2 congenital lipodystrophy caused by *BSCL2* gene mutation, and dietary modification was initiated. Conventional echocardiography performed at the ages of 5, 8, and 14 months showed no significant changes and a normal ejection fraction. However, two-dimensional speckle-tracking performed between the ages of 5 and 8 months showed cardiac systolic abnormalities that tended to improve after treatment. This case highlights the value of echocardiography in detecting structural and early functional cardiac changes in infants with congenital lipodystrophy.

## Introduction

First reported in 1954, congenital lipodystrophy (CGL) is a very rare autosomal recessive disorder with a heterogeneous presentatio n. CGL has an estimated prevalence of about 1/10,000,000 and is generally associated with parental consanguinity (1). The most extreme phenotype of CGL is severe insulin resistance with the loss of nearly all the body fat at birth and early development of metabolic complications in childhood (1,2). Another four distinct genetic subtypes of CGL have been reported to date, which are associated with mutations of *AGPAT2*, *BSCL2*, *CAV1*, and *PTRF*, respectively (3). Besides the common clinical manifestations, patients with type 1 CGL might present with acromegaloid features with an enlarged mandible, hands, and feet, and bone cysts as a late complication. Patients with type 2 CGL have an increased prevalence of cardiomyopathy and mild mental retardation and have lower median serum concentrations of leptin and adiponectin than healthy individuals. Patients with type 3 CGL have serum creatine kinase concentrations between 2.5 and 10 times the upper limit of normal. Patients with type 4 CGL have congenital myopathy with high serum concentrations of creatine kinase and a predisposition to serious arrhythmias (1,2). The most common subtypes of CGL are types 1 and 2, and the cardiac abnormalities in patients with CGL usually occur in childhood or later in adulthood (4). In this report, we describe an infant with type 2 CGL in whom early cardiac involvement was diagnosed by echocardiography.

## Case presentation

**Figure 1:**
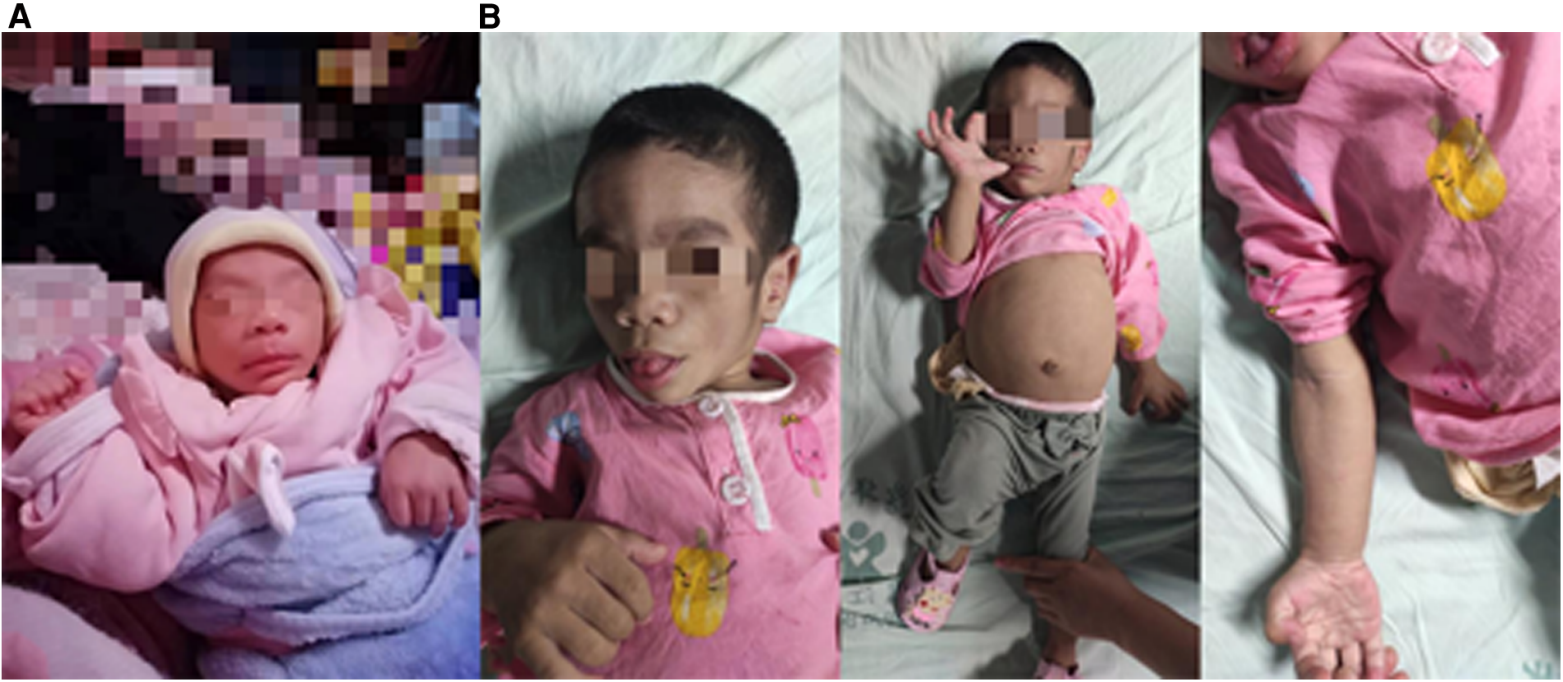
Physical appearance of the patient at the ages of (**A**) 1 month and (**B**) 5 months.

An 8-month-old girl was admitted to the pediatric department of our hospital after first presenting at the age of 3 months with abnormal facial features that had been noticed within 4 weeks of birth (Figure 1A). Her parents were healthy first cousins. Her prenatal ultrasound examination showed no obvious abnormalities. Because the age of the mother at the expected delivery date was more than 35 years, chromosome microarray analysis by amniocentesis had been performed. The chromosome microarray analysis showed no chromosome number abnormalities or pathogenic copy number variation, and detected eight loss of heterozygosity regions of unknown significance. The mother refused whole-exome sequencing of amniotic fluid owing to the high cost. The patient was born after a full-term pregnancy with a birth weight of 2860 g and an Apgar score of 9–10–10. She had no significant family history and had a healthy 9-year-old sister.

**Figure 2:**
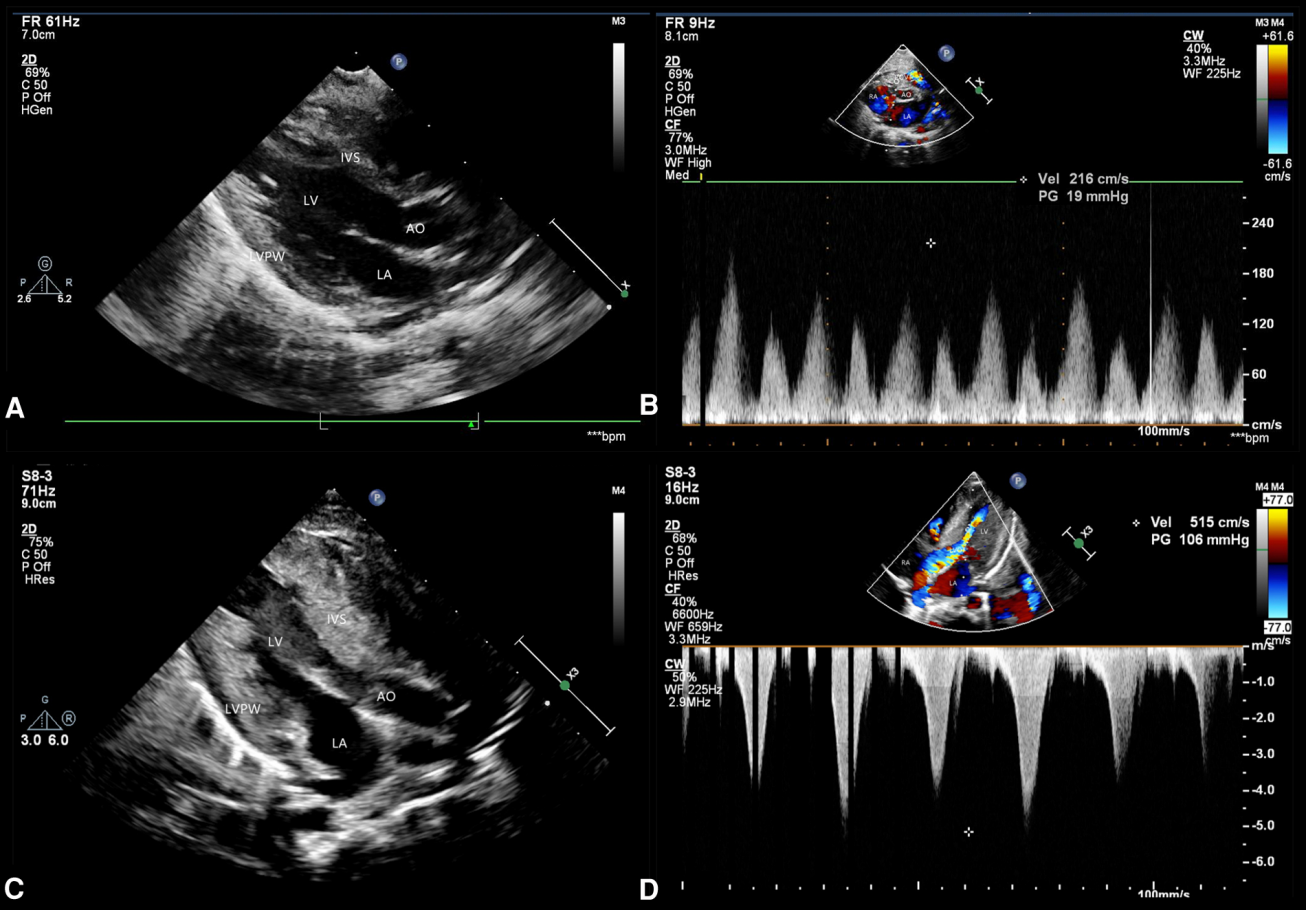
Echocardiographic findings. Echocardiography performed at the age of 3 months shows (**A**) a normal ventricular wall thickness but (**B**) slightly accelerated blood flow in the right ventricular outflow tract. Echocardiography performed at 5 months of age shows (**C**) significant myocardial hypertrophy and (**D**) an obstructive pattern in the left ventricular outflow tract.

At the age of 3 months, physical examination revealed a progeroid facial appearance of coarse facial features with prominent cheekbones and a triangular chin, a low frontal and posterior hairline, and facial hirsutism. She also had hepatomegaly (7 cm in the midclavicular line) with no subcutaneous fat, marked hypertrophy of muscles in the limbs, and acanthosis nigricans. A 3/6 systolic murmur was heard in the precardiac region. Echocardiography showed normal ventricular wall thicknesses (Figure 2A). (interventricular septum (IVS) measured as 4.5 mm, Z score +1.62; left ventricular posterior wall (LVPW) measured as 4 mm, Z score +0.24) and slightly accelerated blood flow in the right ventricular outflow tract (Figure 2B). The preliminary diagnosis was progeria without further treatment.

**Figure 3:**
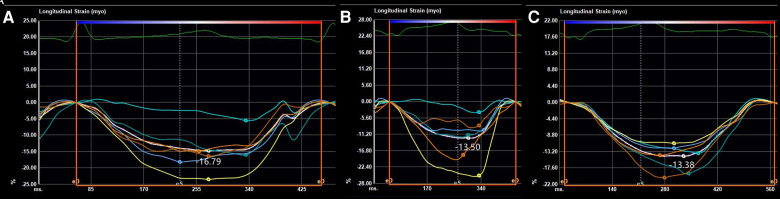
Strain parameters on two-dimensional speckle-tracking of the global longitudinal strain at the ages of (**A**) 5 months, (**B**) 8 months, and (**C**) 14 months.

**Figure 4:**
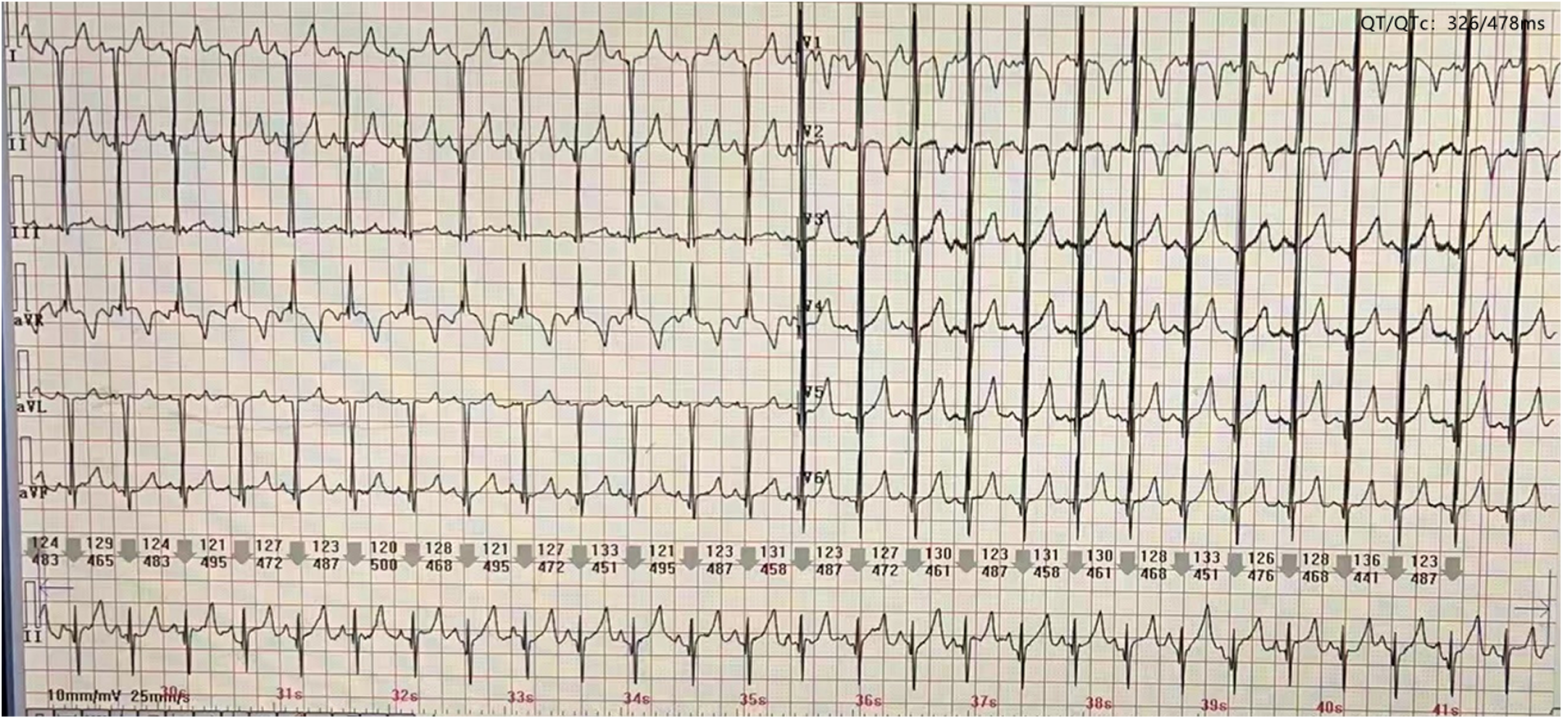
Electrocardiography reveals a prolonged QTc interval.

At the age of 5 months, the physical examination findings were similar to the findings at 3 months (Figure 1B). Standard conventional echocardiography performed at the age of 5 months showed myocardial hypertrophy (Figure 2C). (IVS measured as 13 mm, Z score +23.21; LVPW measured as 8.5 mm, Z score +9.1) and an obstructive pattern in the left ventricular outflow tract (Figure 2D) but a normal ejection fraction of 70%. Because the ventricular wall thickness was significantly thicker at the age of 5 months than it was at the age of 3 months, we ruled out the diagnosis of classic hypertrophic cardiomyopathy and considered that the ventricular wall hypertrophy was caused by metabolic factors. Two-dimensional speckle-tracking (2D-STE) was performed to assess strain parameters in the left ventricle and revealed a global longitudinal strain of −16.79% (Figure 3A), a global radial strain of 65.67%, and a global circumferential strain of −14.04%. Abdominal ultrasound showed hepatic steatosis with hepatomegaly. An electrocardiogram revealed sinus rhythm and a prolonged QTc interval (Figure 4). Her blood pressure was 123/65 mmHg. Additional fasting blood testing showed an increased free fatty acid concentration (1.39 mmol/L), normoglycemic hyperinsulinemia (glycemia concentration, 4.60 mmol/L; insulin concentration, 58.53 µIU/mL), dyslipidemia (increased triglycerides [10.14 mmol/L], total cholesterol [5.33 mmol/L], and low-density lipoprotein cholesterol [3.8 mmol/L]), and decreased high-density lipoprotein cholesterol (0.69 mmol/L) indicating insulin resistance. CGL was considered based on the physical characteristics and investigations.

At the age of 8 months, 2D-STE showed a decrease in several left ventricular strain parameters (global longitudinal strain, −13.50% (Figure 3B); global radial strain, 37.63%; and global circumferential strain, −14.76%) compared with the values recorded at the age of 5 months despite the findings on conventional echocardiography showing no significant change (IVS measured as 13.5 mm, Z score +23.34; LVPW measured as 8.5 mm, Z score +9.7). Combined with the typical clinical manifestations (severe insulin resistance with lipoatrophy and hypertrophic cardiomyopathy, and hirsutism), exon group of single gene disease-customized capture sequencing test at the age of 9 months identified a homozygous mutation in the *BSCL2* gene: c.166_184del (*p*.Tyr56fs*) (reference sequence NM_032667.6) on 11q12.3, confirming a diagnosis of type 2 CGL (OMIM # 269700).. Both her mother and sister were confirmed to be heterozygous carriers. Dietary modification was introduced to restrict her fat intake to 25% of the total dietary energy. Follow-up biochemistry at the age of 14 months showed progressive improvement in the fasting lipid profile (triglycerides, 4.13 mmol/L; total cholesterol, 5.10 mmol/L; insulin concentration, 29.49 µIU/mL) with normal glycemia concentration [4.5 mmol/L]. Her blood pressure at the age of 14 months was normal. Conventional echocardiography showed no significant changes (IVS measured as 13 mm, Z score +20.55; LVPW measured as 8.5 mm, Z score +7.75) from the findings at the age of 8 months, and the left ventricular strain parameters measured by 2D-STE tended to remain stable (global longitudinal strain, −13.38% (Figure 3C); global radial strain, 34.60%; global circumferential strain, −14.41%).

## Discussion

A diagnosis of CGL is based on three major criteria or two major criteria plus more than two minor criteria and/or genetic testing (2). The major criteria are: lipoatrophy affecting the trunk, limbs, and face; acromegaloid features; hepatomegaly; elevated serum triglycerides; and insulin resistance. The minor criteria are hypertrophic cardiomyopathy, psychomotor retardation or mild to moderate intellectual impairment, hirsutism, precocious puberty (female), bone cysts, and phlebomegaly.

Although cardiac involvement in CGL was first reported in 1959 (5), the pathophysiological mechanism is still not completely clear; however, it may involve the combined effects of abnormal metabolism, dysfunction in the autonomic nervous system, and reduction or loss of epicardial adipose tissue (4, 6). A variety of cardiac problems have been described, ranging from cardiomyopathy, impaired cardiac function, myocardial infarction, and arrhythmia to heart failure and sudden death (2, 3). Hypertrophic cardiomyopathy is reported to be present in 20%–25% of individuals with CGL (2), is found mainly in type 2 but occasionally in type 1 (4), and is a significant cause of morbidity associated with cardiac failure and with early mortality at around 30 years of age (2). Arrhythmias are mostly seen in type 4 CGL (3), while a long QT interval is commonly seen in types 2 and 4 (4). QT prolongation is an independent predictor of all-cause and cardiovascular mortality (3).

The reported mean age at the time of diagnosis of cardiomyopathy in patients with CGL is 20 years (7), with cardiovascular manifestations occurring earlier in type 2 CGL (4). Most of the published data on cardiac involvement in infants with CGL come from case reports, in which hypertrophic cardiomyopathy is the most common cardiac manifestation (7–11). The literature includes the following cases: a boy with type 1 CGL in whom concentric thickening of the left ventricle, a mild obstructive pattern in the left ventricular outflow tract, and tachycardia were noted at 2 months of age (7) and four infants with type 2 CGL (a girl who showed asymmetric hypertrophic cardiomyopathy at the age of 6 months (8), a boy with asymmetric hypertrophic cardiomyopathy, an obstructive pattern in the left ventricular outflow tract, and tachycardia at 4 months of age (9), a boy who showed hypertrophic cardiomyopathy at the age of 4 months and worsening of left ventricular wall thickness at the age of 6.5 months (10), and a boy who was mentioned to have cardiomyopathy at 2 months of age with no further clinical details provided) (11). In our case, the patient was initially diagnosed with progeria, but the cardiac changes and unusual clinical features led us to consider the diagnosis of CGL. Cardiac involvement was first detected as slightly accelerated blood flow in the right ventricular outflow tract at the age of 3 months that developed into hypertrophic cardiomyopathy combined with a prolonged QTc interval by the age of 5 months. To our knowledge, this is the first report in which echocardiography observed the thickening process of the ventricular wall from normal thickness to asymmetric hypertrophy, and the simultaneous occurrence of cardiomyopathy and this type of arrhythmia has not been reported previously in infants with type 2 CGL. However, the slightly accelerated blood flow in the right ventricular outflow tract may have resulted from slight myocardial hypertrophy of the basal anterior septum, which may have been ignored during the examination because the basal segment of the anterior ventricular septum is more likely to be involved in patients with CGL (12). Furthermore, using 2D-STE, we observed progressive decreases in global longitudinal strain and global radial strain between the ages of 5 and 8 months with a normal ejection fraction. This finding suggests subclinical impairment of myocardial systolic function and is in accordance with the results of the only previous study that has used 2D-STE to detect cardiac alterations in patients with CGL (13). A possible explanation of these findings is the histopathological changes that occur in these patients, including diffuse distribution of interstitial deposits of collagen, severe subendocardial fibrosis, hypertrophy of myocytes, and coronary atherosclerosis (14). The higher sensitivity of 2D-STE allows early detection of cardiac systolic abnormalities, and our finding that 2D-STE parameters tended to improve when dietary modification was introduced into the treatment plan confirms that metabolic abnormalities contribute to cardiac involvement in CGL. Therefore, we recommend 2D-STE for early evaluation of myocardial dysfunction in children with CGL and for decision-making regarding an appropriate treatment strategy. If the patient's 2D-STE parameters are improved after just restricting the total fat intake, there is no need to use drugs; however, once the cardiac function starts worsening, even if the ejection fraction is normal, we recommend the addition of drugs such as leptin.

## Conclusion

We have encountered a case of cardiac involvement in an infant with type 2 CGL as a result of *BSCL2* mutation. Echocardiography is helpful for real-time detection of cardiac changes in patients with CGL and can be used for follow-up examinations. This is the first report of the simultaneous occurrence of cardiomyopathy and a prolonged QTc interval in an infant with CGL, and the thickening process of ventricular wall was observed by echocardiography. There is only one previous report on the use of 2D-STE for the detection of cardiac alterations in patients with CGL, with our case report being the first report to use 2D-STE to detect cardiac alterations and monitor myocardial function in an infant.

## Data Availability

The original contributions presented in the study are included in the article/Supplementary Material, further inquiries can be directed to the corresponding author.
